# Generalisation of genomic findings and applications of polygenic risk scores

**DOI:** 10.1186/s12920-023-01615-7

**Published:** 2023-07-28

**Authors:** Manuel Corpas, Segun Fatumo

**Affiliations:** 1 Life Sciences, University of Westminster, 115 New Cavendish Street, London, UK; 2 Cambridge Precision Medicine Limited, ideaSpace, University of Cambridge Biomedical Innovation Hub, Cambridge, UK; 3 African Computational Genomics (TACG) Research Group, MRC/UVRI and LSHTM, Research Unit, Entebbe, Uganda; 4 Department of Non-Communicable Disease Epidemiology (NCDE), London School of Hygiene and Tropical Medicine, London, UK

## Abstract

Polygenic Risk Scores (PRS) (also known as polygenic scores, genetic risk scores or polygenic indexes) capture genetic contributions of a multitude of markers that characterise complex traits. Although their likely application to precision medicine remains to be established, promising advances have included their ability to stratify high risk individuals and targeted screening interventions. Current PRS have been mostly optimised for individuals of Northern European ancestries. If PRS are to become widespread as a tool for healthcare applications, more diverse populations and greater capacity for derived interventions need to be accomplished. In this editorial we aim to attract submissions from the research community that highlight current challenges in development of PRS applications at scale. We also welcome manuscripts that delve into the ethical, social and legal implications that the implementation of PRS may generate.

Throughout the history of the Human Genome Project [1], one of its expected outcomes was the provision of predictive models and mechanisms to diagnose disease early, preferably before symptoms arise. Polygenic Risk Scores (PRS) are a promising recent development that bridges the expected outcome of the Human Genome Project to help predict disease at early stages. PRS applications not only include disease prevention, they also can be applied to improve clinical management, drug development, population risk stratification and targeted screening interventions [2, 3]. PRS may also be helpful for better understanding of the underlying genetic mechanisms of a particular disease [4].

Many challenges become apparent as a consequence of wide implementation of PRS [3]. Firstly, PRS have mostly been trained with data from Northern European populations [5, 6]. Because PRS represent most accurately those populations from which they have been trained, the further the training dataset from the test dataset, the less accurate they are for predicting traits in a different population [7]. However, despite recent studies calling for more diversity in PRS models, the gap continues to widen [8]. Public repositories have been developed to provide indexing and access to published PRS [9, 10], yet it can be very confusing to choose a particular PRS for testing, as there is a lot of overlap that exists between PRS representing the same or similar phenotypes. It does not help either that there are slightly different reporting and accuracy metrics (depending on the source study) and that some of the publicly available PRS are under a licence which makes them challenging for their reutilisation under certain circumstances [11].

Implementation of PRS at scale in health services, therefore, remains a challenge, particularly the translation of PRS into actionable benefits for the individual. Internationally leading initiatives such as ‘Our Future Health’ [12] have the ambition to use PRS in healthcare settings for their application to the wider population. Such application, however, requires of standard study designs to include diverse populations and a systematic deployment of PRS into actionable outcomes for the patient.

Our Collection on “Generalisation of Genomic Findings and Applications of Polygenic Risk Scores” aims to attract original research or systematic reviews that address some of the aforementioned challenges. We are particularly interested in new insights or solutions into the implementation or integration of PRS within healthcare systems. Topics sought after also include (but are not restricted to) the impact of ancestry in PRS accuracy, the transferability of PRS to underrepresented populations, their integration with other data sources that help stratify and predict high risk individuals, the translation of PRS into actionable outcomes and interventions for individuals within a health system. We welcome too ethical, social implications derived from the implementation of PRS on a wide scale within national or private health delivery systems.

We expect advances reported in this collection to improve the current implementation at scale of PRS in both private and public health systems. Such research is expected to empower the next generation of genetic testing aiming to make use of PRS as an essential tool for reporting genetic risk [13, 14]. We suggest that for PRS to be applied at scale, several important developments are needed to be advanced: (a) to further the collection of diverse data through biobanks, (b) the development of clinical trials making use of PRS and (c) the generation of public/private partnerships with commercial genetic testing companies. For this, the research community will need to use and develop new bioinformatics methods and machine learning approaches in order to analyse and generate PRS data [15]. PRS will need to be integrated with electronic health records to provide clinicians with information about an individual’s health risks. Higher learning organisations will be required to provide education to patients about the meaning and limitations of PRS as well as the benefits and risks when integrated as part of preventative medicine. Healthcare providers will also need to be trained to provide counselling to patients and their families so that PRS-based genetic risk is properly conveyed, communicated and acted upon [16]. PRS implementations in a healthcare context will need to consider ethical and legal implications of implementing PRS at scale, including issues related to patient privacy, informed consent and genetic discrimination. Finally, new initiatives and health systems that include PRS in their future plans should also consider to continuously evaluate and monitor the impact of PRS on patient outcomes as well as the cost-effectiveness of implementing PRS at scale.

## Data Availability

No data were analysed for this manuscript. All materials used have been referenced.
